# Supplementary material for: PRMT3-mediated arginine methylation of IGF2BP1 promotes oxaliplatin resistance in liver cancer
Source: Nat Commun. 2023 Apr 6;14:1932. doi: 10.1038/s41467-023-37542-5 (PMC10079833; doi:10.1038/s41467-023-37542-5)
Supplement: Supplementary file 1 — Supplementary Information [file 41467_2023_37542_MOESM1_ESM.pdf]

Supplementary information

PRMT3-mediated arginine methylation of IGF2BP1 promotes oxaliplatin resistance in liver cancer

Yunxing Shi, Yi Niu, Yichuan Yuan, Kai Li, Chengrui Zhong, Zhiyu Qiu, Keren Li, Zhu Lin, Zhiwen Yang, Dinglan Zuo, Jiliang Qiu, Wei He, Chenwei Wang, Yadi Liao, Guocan Wang, Yunfei Yuan, Binkui Li

Supplementary figures

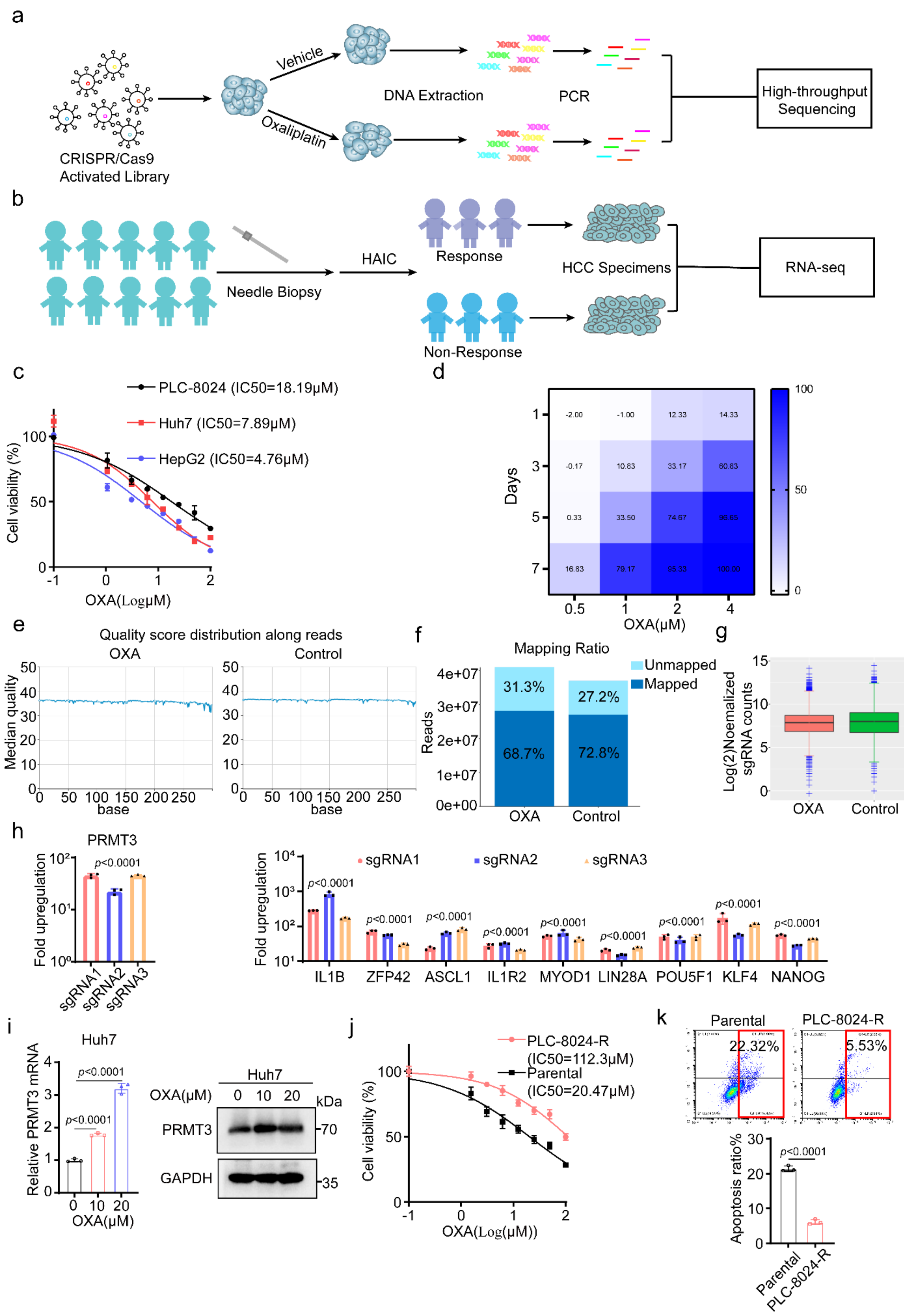

Supplementary Fig. 1 CRISPR/Cas9 activation library screen and transcriptome analysis of patient samples identify PRMT3 as a candidate driver for OXA resistance in HCC

a Schematic diagram illustrates the workflow of genome-wide CRISPR/Cas9 activation library screen.

b Schematic diagram illustrates the workflow of clinical HCC sample (Response VS Non-response to HAIC) screening.

c The IC50 of OXA in PLC-8024, Huh7 and HepG2 cells.

d The effect of different concentrations of OXA on proliferation inhibition ratio in hepG2 cells.

e The measurements of median base quality.

f The measurements of total number of reads and the percentage of mapped reads.

g The measurements of normalized read count distribution.

h The mRNA expressions of target genes in HepG2 cells transferred with indicated sgRNAs or control. Data were shown as fold change normalized to the control group.

i The mRNA and protein level of PRMT3 in PLC-8024 cells treated with OXA (0, 25, and 50 μM) for 48h (n=3 independent experiments for Western blot assay).

j The IC50 of OXA in PLC-8024 parental and PLC-8024-R cells.

k Apoptosis of PLC-8024 parental and PLC-8024-R cells treated with OXA (40 μM) using flow cytometry analysis of Annexin V staining.

For **c**, **h**, **i** and **k**, n=3 biologically independent samples. For **j**, n=5 biologically independent samples. Data in **c**, **h**, **i**, **j** and **k** are presented as mean ± SD. All Box plot center line represents

median value; lower and upper hinges represent 25th and 75th percentiles; the minimum and maximum are indicated by the extremes of the box plot. Data were analyzed by two-sided Student's t test in **h**, **i** and **k**. Source data are provided as a Source Data file.

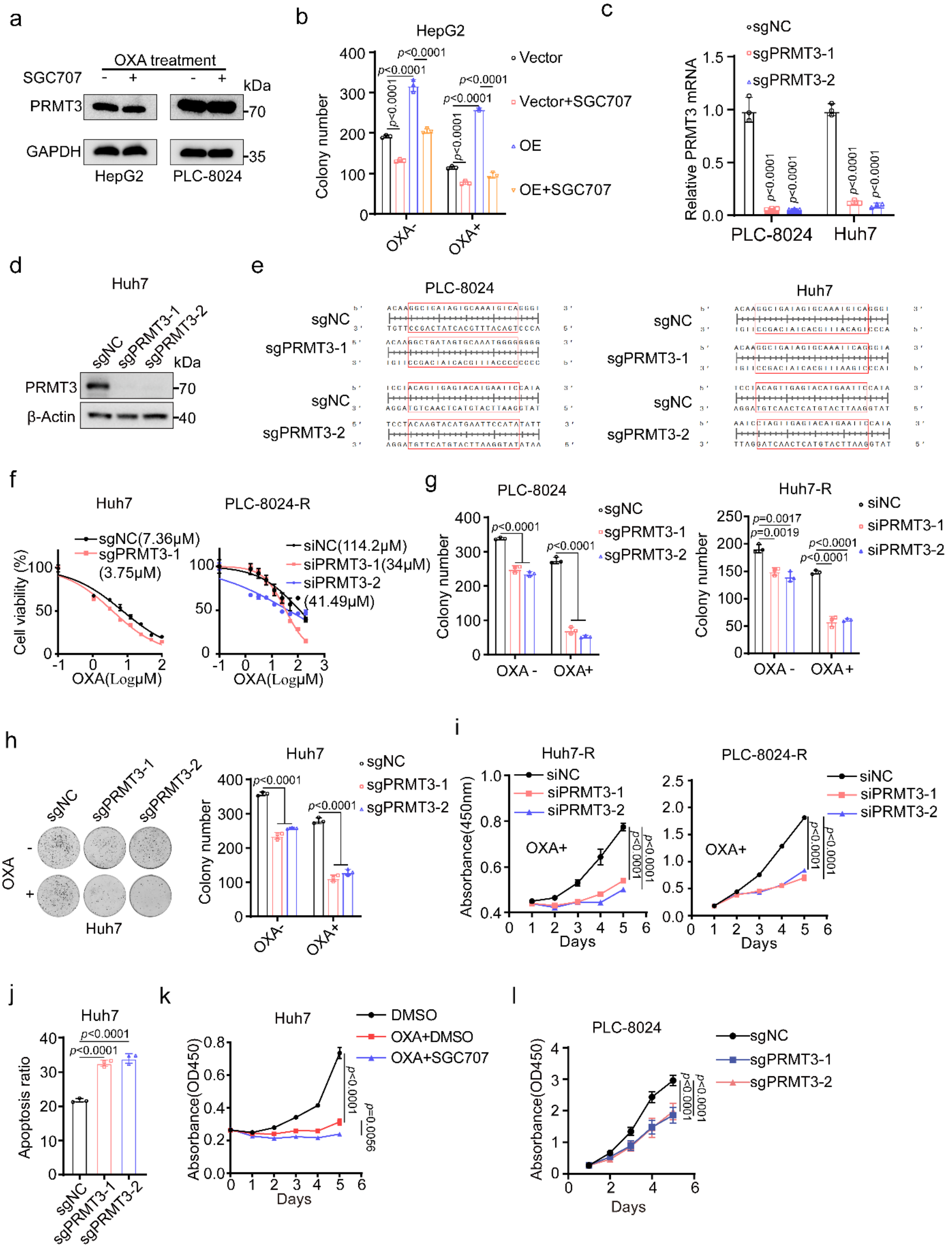

a
OXA treatment
SGC707
- + - +
kDa
PRMT3
-70
GAPDH
-35
HepG2
PLC-8024
b
HepG2
Colony number
p<0.0001
Vector
Vector+SGC707
OE
OE+SGC707
OXA-
OXA+
c
sgNC
sgPRMT3-1
sgPRMT3-2
Relative PRMT3 mRNA
p<0.0001
PLC-8024
Huh7
d
Huh7
sgNC
sgPRMT3-1
sgPRMT3-2
kDa
PRMT3
-70
β-Actin
-40
e
PLC-8024
Huh7
sgNC
sgPRMT3-1
sgPRMT3-2
f
Huh7
PLC-8024-R
sgNC(7.36μM)
sgPRMT3-1 (3.75μM)
siNC(114.2μM)
siPRMT3-1(34μM)
siPRMT3-2 (41.49μM)
Cell viability (%)
OXA(LogμM)
g
PLC-8024
Huh7-R
sgNC
sgPRMT3-1
sgPRMT3-2
siNC
siPRMT3-1
siPRMT3-2
Colony number
p<0.0001
p=0.0017
p=0.0019
OXA -
OXA+
h
sgNC
sgPRMT3-1
sgPRMT3-2
OXA
-
+
Huh7
Colony number
p<0.0001
OXA-
OXA+
i
Huh7-R
PLC-8024-R
siNC
siPRMT3-1
siPRMT3-2
OXA+
Absorbance(450nm)
p<0.0001
Days
j
Huh7
Apoptosis ratio
p<0.0001
sgNC
sgPRMT3-1
sgPRMT3-2
k
Huh7
DMSO
OXA+DMSO
OXA+SGC707
Absorbance(OD450)
p<0.0001
p=0.0056
Days
l
PLC-8024
sgNC
sgPRMT3-1
sgPRMT3-2
Absorbance(OD450)
p<0.0001
Days

Supplementary Fig. 2 PRMT3 promotes OXA resistance in vitro and in vivo

a Western blot analysis of PRMT3 expression in HCC cells treatment with OXA and/or SGC707.

b The effects of PRMT3 overexpression and inhibition of PRMT3 by SGC707 on the growth of HepG2 cells in the presence and absence of OXA treatment (0.1 μM) as shown by colony-formation assay.

c, d PRMT3 expression as shown by qPCR (**c**) /Western blot (**d**) analysis in PRMT3-KO cells and control cells.

e Sequence alignment of sgRNA targeted DNA of *PRMT3* KO and control PLC-8024/Huh7 cells.

f The IC50 of OXA in PRMT3 KO/KD and control Huh7/PLC-8024-R cells.

g The effects of PRMT3 KO/KD on the growth of PLC-8024 and Huh7-R cells in the presence and absence of OXA treatment (0.5 μM) as shown by colony-formation assay.

h The effects of PRMT3 KO on the growth of Huh7 cells in the presence and absence of OXA treatment (0.5 μM) as shown by colony-formation assay.

i The effects of PRMT3 KD on the growth (0.5 μM OXA) of PLC-8024-R and Huh7-R cells as shown by CCK8 assay.

j The effects of PRMT3 KO on the apoptosis (40 μM OXA) of Huh7 cells using flow cytometry analysis.

k CCK8 assay to measure the effects of PRMT3 inhibitor, SGC707 (100 μM), on cell proliferation (1 μM OXA) in Huh7.

l CCK8 assay to measure the effects of PRMT3 KO on cell proliferation in PLC-8024 cells.

For **b**, **c, f-h** and **j,** n=3 biologically independent samples. For **i** and **k**, n=5 biologically independent samples. For **l**, n=6 biologically independent samples. For **a** and **d**, n=3 independent experiments. Data in **b**, **c**, **f-l** are presented as mean ± SD. Data were analyzed by two-sided Student's t test in **b**, **c**, **g-l**. Source data are provided as a Source Data file.

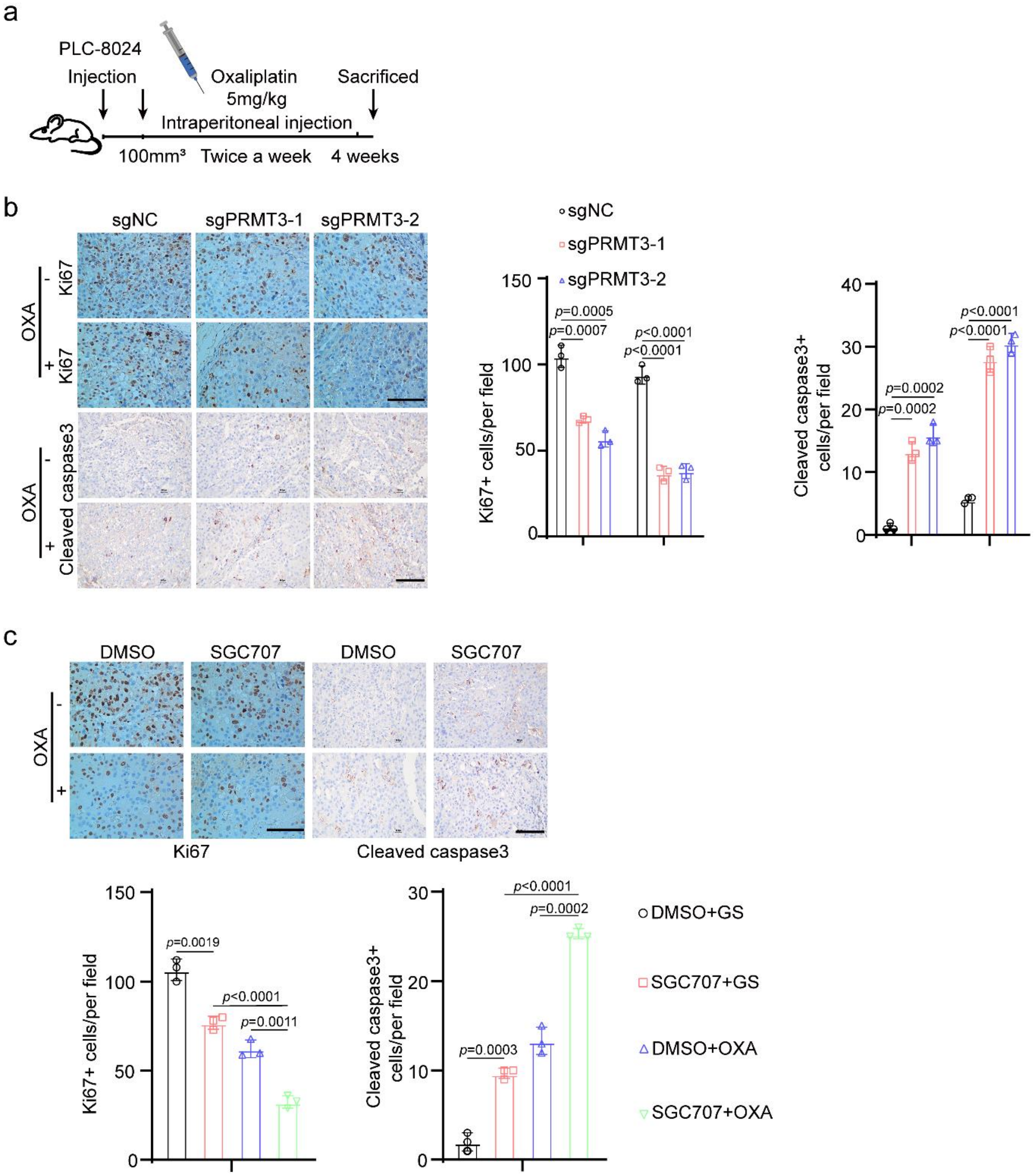

Supplementary Fig. 3 PRMT3 promotes OXA resistance in vitro and in vivo

a Schematic diagram of the subcutaneous transplantation HCC model treated with SGC707 and/or OXA.

b IHC staining for Ki67 and cleaved Caspase 3 in tumors from subQ implanted PRMT3 KO PLC-8024 cells and control cells treated with OXA or vehicle. Scale bars,100 μm.

c IHC staining for Ki67 and cleaved Caspase 3 in tumors from subQ implanted PLC-8024 cells

treated with vehicle, OXA (5mg/kg), SGC707 (20mg/kg), and OXA+SGC707.

For **b** and **c**, n=3 biologically independent samples. Data in **b** and **c** are presented as mean ± SD. Data were analyzed by two-sided Student's t test in **b** and **c**. Source data are provided as a Source Data file.

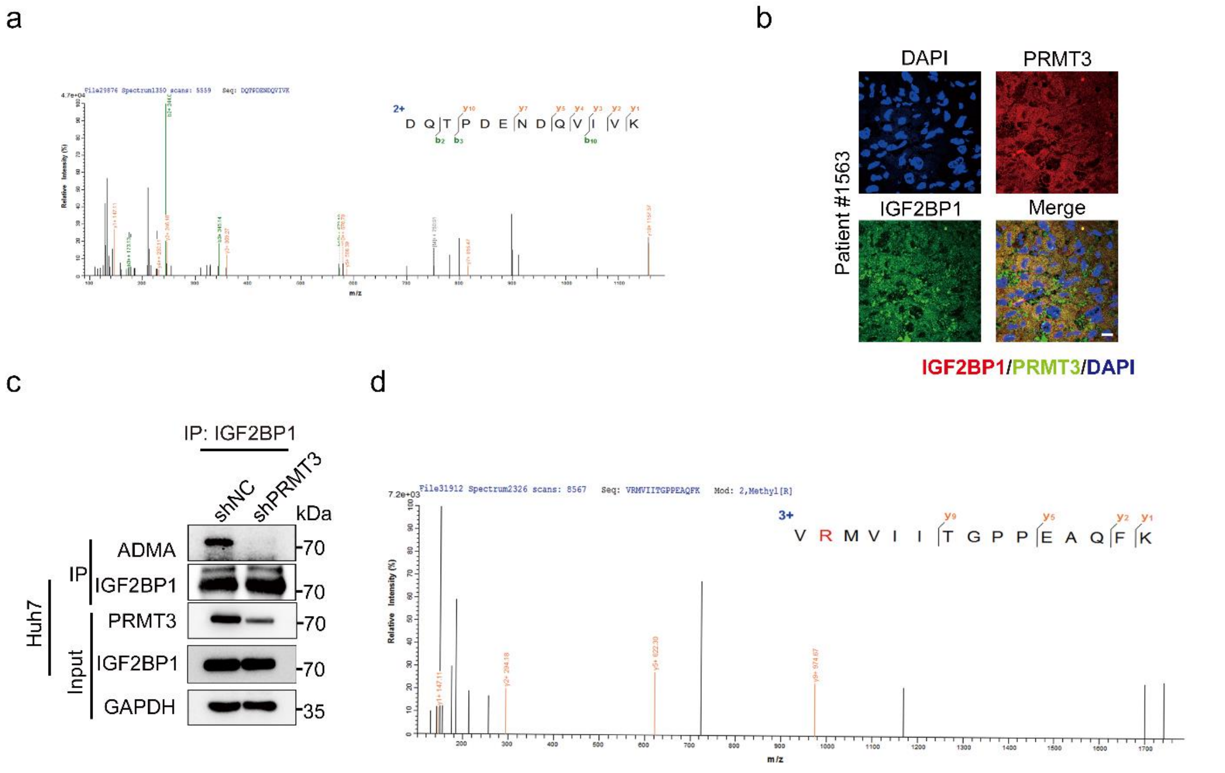

Supplementary Fig. 4 PRMT3 methylates IGF2BP1 at R452

a Fragmentation spectrum of peptides identified by liquid chromatography/tandem mass spectrometry (LC-MS/MS).

b Immunofluorescence staining showed the co-localization of PRMT3 (green) and IGF2BP1 (red) in HCC patient samples. Scale bar, 50 μm.

c WB analysis of immunoprecipitated IGF2BP1 to determine the effect of PRMT3 KD on arginine methylation of IGF2BP1 in Huh7 cells.

d Fragmentation spectrum of the methylated peptide identified by liquid chromatography/tandem mass spectrometry (LC-MS/MS).

For **b** and **c**, n=3 independent experiments. Source data are provided as a Source Data file.

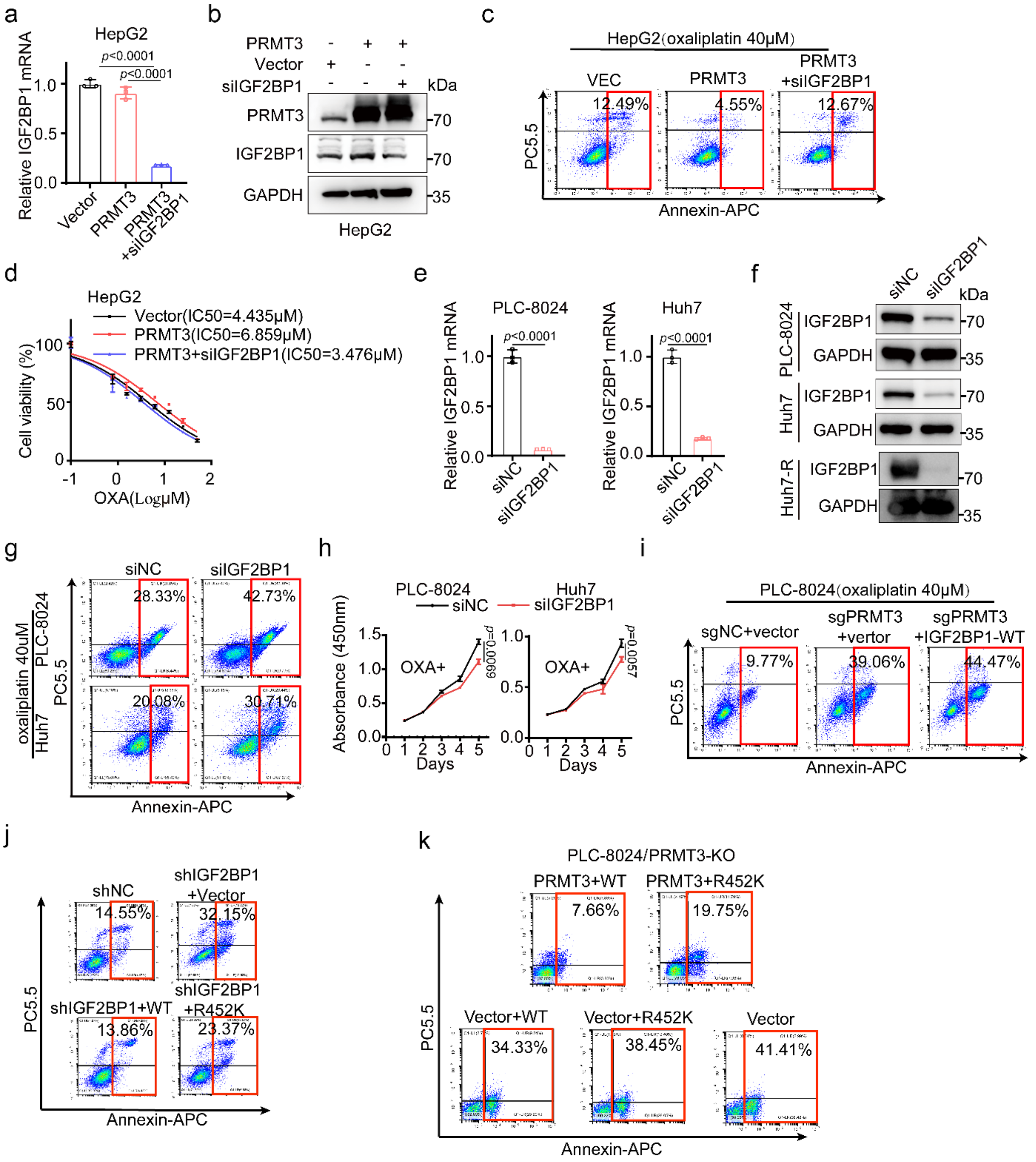

Supplementary Fig. 5 R452 methylation of IGF2BP1 is required for OXA resistance

a, b *IGF2BP1* expression as shown by qPCR and Western blot analysis in PRMT3-overexpressing HepG2 cells transfected with the IGF2BP1 siRNA or corresponding control.

c The effect of IGF2BP1 knockdown on apoptosis as shown by flow cytometry analysis of Annexin V staining in PRMT3-overexpression HepG2 cells treated with OXA (40 μM).

d The IC50 of OXA in PRMT3-overexpressing HepG2 cells transfected with the IGF2BP1 siRNA or corresponding control.

e, f IGF2BP1 expression as shown by qPCR and Western blot analysis in IGF2BP1 knockdown

cells and control cells.

g The effect of IGF2BP1 knockdown on apoptosis as shown by flow cytometry analysis of Annexin V staining in PLC-8024 and Huh7 cells treated with OXA (40 μM).

h CCK8 assay to measure the effects of IGF2BP1 knockdown on cell proliferation in PLC-8024 and Huh7 cells.

i The effect of IGF2BP1-WT OE and vector control on apoptosis of PRMT3 knockout PLC-8024 cells treated with OXA (40 μM) using flow cytometry analysis of Annexin V staining.

j The effect of IGF2BP1-WT and IGF2BP1-R452K mutant OE on apoptosis of IGF2BP1-KD PLC-8024 cells and control cells treated with OXA (40 μM) as shown by flow cytometry.

k The effect of IGF2BP1-WT and R452K mutant OE on apoptosis of PRMT3-KO PLC-8024 cells and PRMT3-KO PLC-8024 cells with PRMT3 OE in the presence of OXA treatment (40 μM).

For **a**, **c**, **d**, **e**, **g-k**, n=3 biologically independent samples. For **b** and **f**, n=3 independent experiments. Data in **a**, **d**, **e** and **h** are presented as mean ± SD. Data were analyzed by two-sided Student's t test in **a**, **e** and **h**. Source data are provided as a Source Data file.

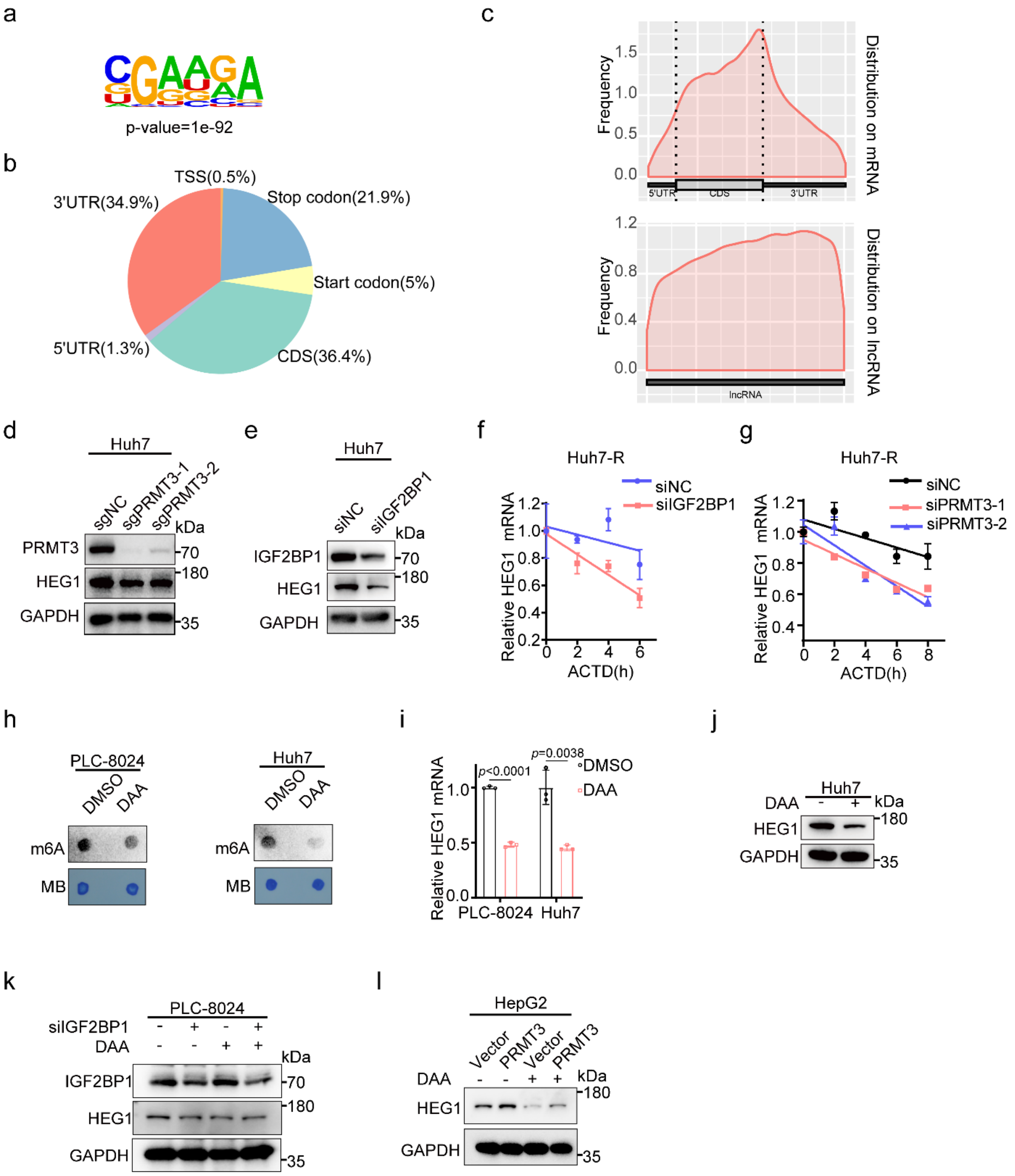

Supplementary Fig. 6 PRMT3 and IGF2BP1 regulate HEG1 expression in an m6A-independent manner

a The m6A consensus sequence motif in the HCC cells.

b, c Distribution of m6A modification in mRNA and lncRNA transcripts. The m6A signal is mostly enriched in the coding sequences.

d WB analysis of HEG1 in PRMT3-KO and control Huh7 cells.

e WB analysis of HEG1 in IGF2BP1-KD and control Huh7 cells.

f The effect of IGF2BP1 KD on the stability of *HEG1* mRNA.

g The effect of PRMT3 KO on the stability of *HEG1* mRNA.

h mRNAs isolated from HCC cells treated with DMSO or DAA and used in dot blot analyses with an anti-m6A antibody, and MB (methylene blue) staining served as the loading control.

i *HEG1* expression as shown by qPCR in PLC-8024 and Huh7 cells under the treatment of 50 μM DAA.

j HEG1 protein expression in Huh7 cells treated with 50 μM 3-deazaadenosine (DAA) or vehicle.

k HEG1 protein expression in IGF2BP1 knockdown cells and control cells treated with 50 μM DAA or vehicle.

l HEG1 protein expression in PRMT3-overexpressing cells and control cells treated with 50 μM DAA or vehicle.

For **f**, **g** and **i**, n=3 biologically independent samples. For **d, e, h** and **j-l**, n=3 independent experiments. Data in **f, g** and **i** are presented as mean ± SD. Data were analyzed by two-sided Student's t test in **i**. Source data are provided as a Source Data file.

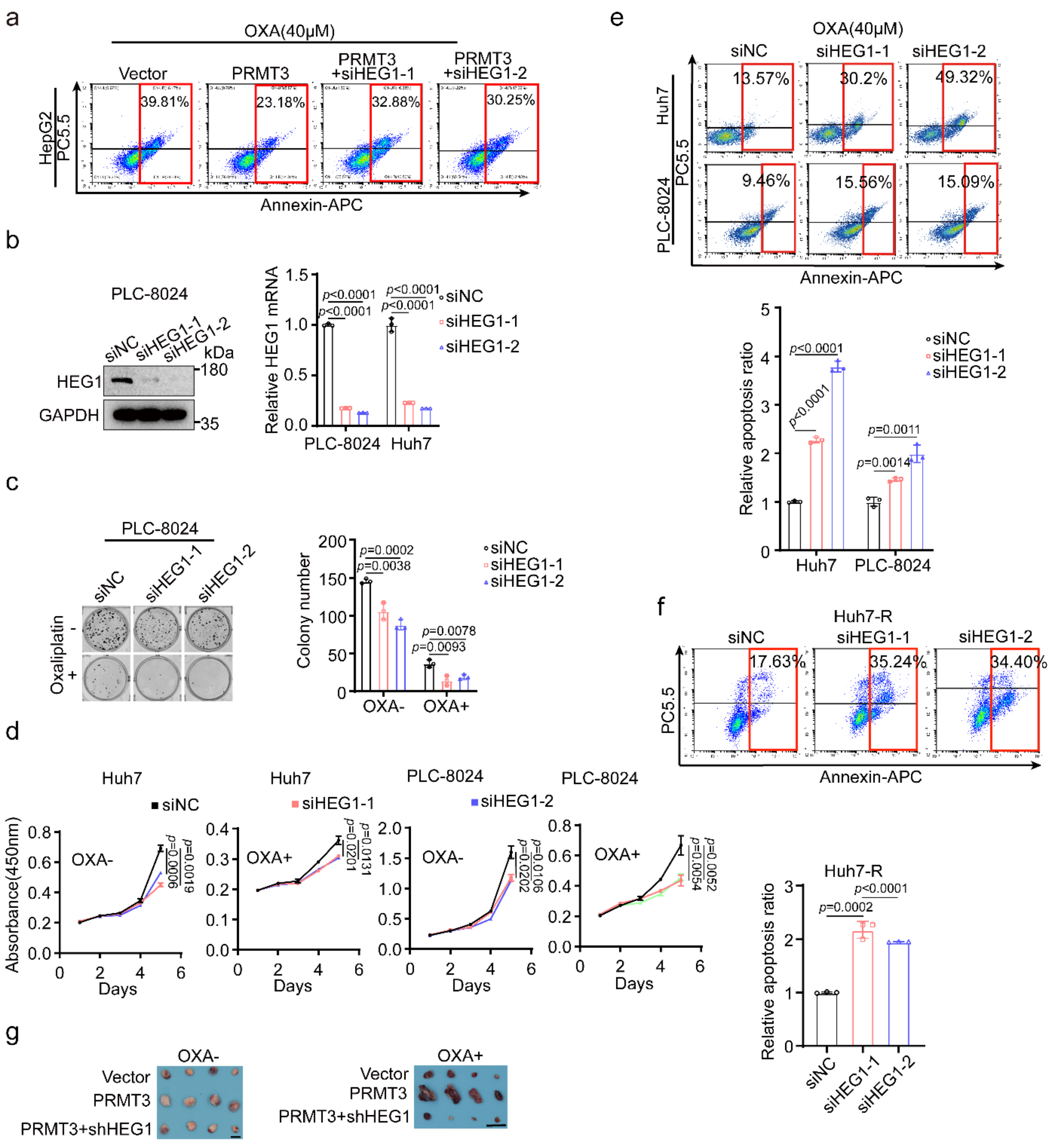

Supplementary Fig. 7 The effect of PRMT3 and IGF2BP1 on OXA resistance is dependent on HEG1 expression

a The effect of HEG1 KD on the apoptosis of HepG2 cells overexpressing PRMT3 treated with OXA (40 μM) as shown by flow cytometry analysis of Annexin V staining.

b HEG1 expression as shown by qPCR and Western blot analysis in HEG1 knockdown cells and control cells.

c The effects of HEG1 knockdown on the growth of HepG2 cells in the presence and absence of OXA treatment (0.5 μM) as shown by colony-formation assay.

d The effect of HEG1 KD on the proliferation of PLC-8024 and Huh7 cells treated with OXA (1 μM) or vehicle as shown by CCK8 assay.

e The effect of HEG1 KD on the apoptosis of PLC-8024 and Huh7 cells treated with OXA (40 μM) as shown by flow cytometry analysis of Annexin V staining.

f The effect of HEG1 KD on the apoptosis of Huh7-R cells treated with OXA (40 μM) as shown by flow cytometry analysis of Annexin V staining.

g The effect of HEG1 knockdown on the tumor growth of subcutaneously implanted HepG2 cells overexpressing PRMT3 treated with oxaliplatin or vehicle (n=6). Scale bars, 1 cm.

For **a**-**f**, n=3 biologically independent samples. For western blot assay in **b**, n=3 independent experiments. Data in **b**-**f** are presented as mean ± SD. Data were analyzed by two-sided Student's t test in **b**-**f**. Source data are provided as a Source Data file.

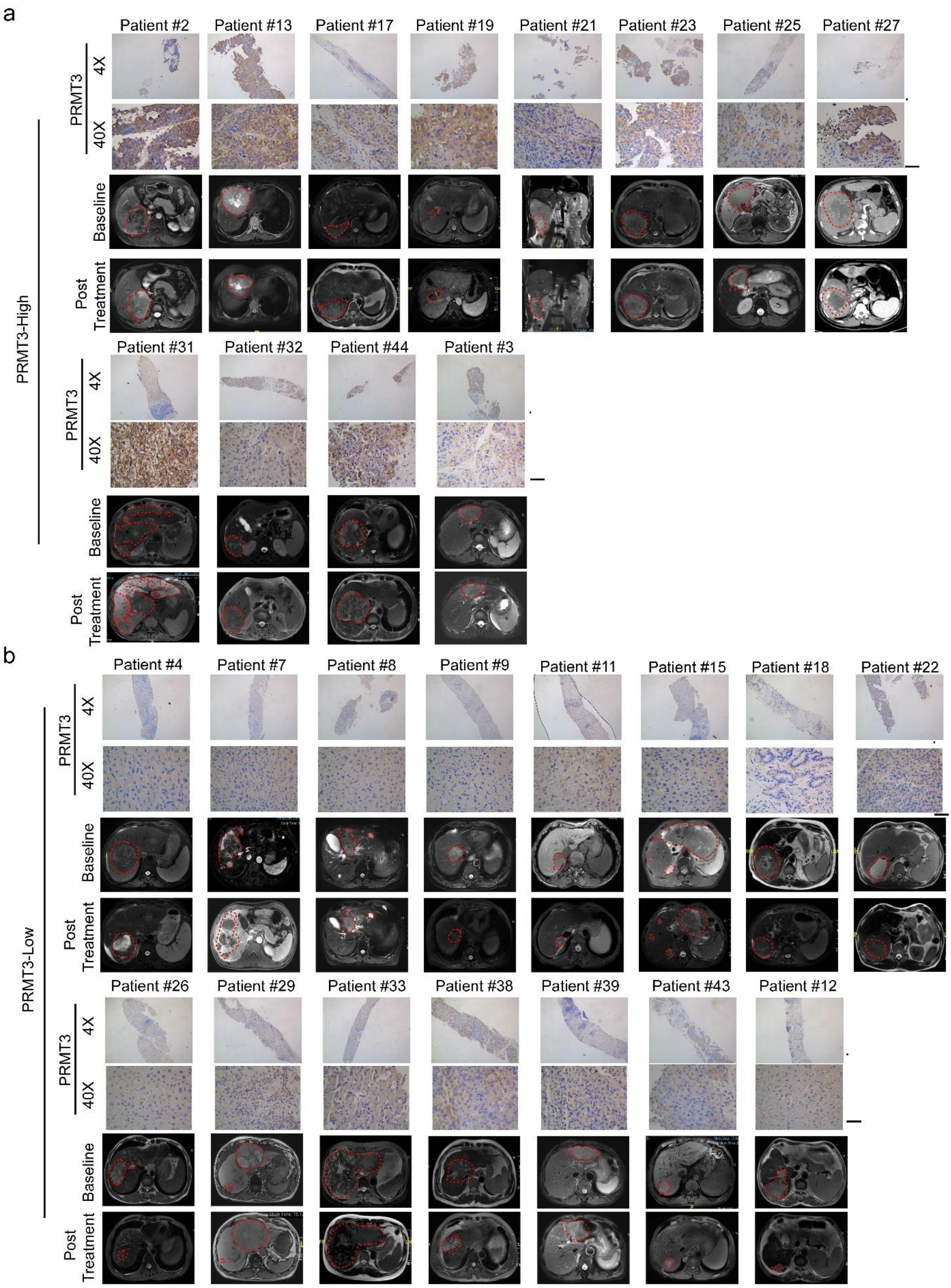

Supplementary Fig. 8 High PRMT3 expression correlates with poor clinical outcomes and poor therapeutic responses to OXA-based HAIC in HCC patients

a, b The baseline and post-treatment MRI images of HCC patients, who had low (n=12 patients) and high (n=15 patients) PRMT3 expression as determined by IHC staining, respectively, showed the patients' response to the OXA-based HAIC Scale bar, 50 μm. The results are representative of three independent experiments.

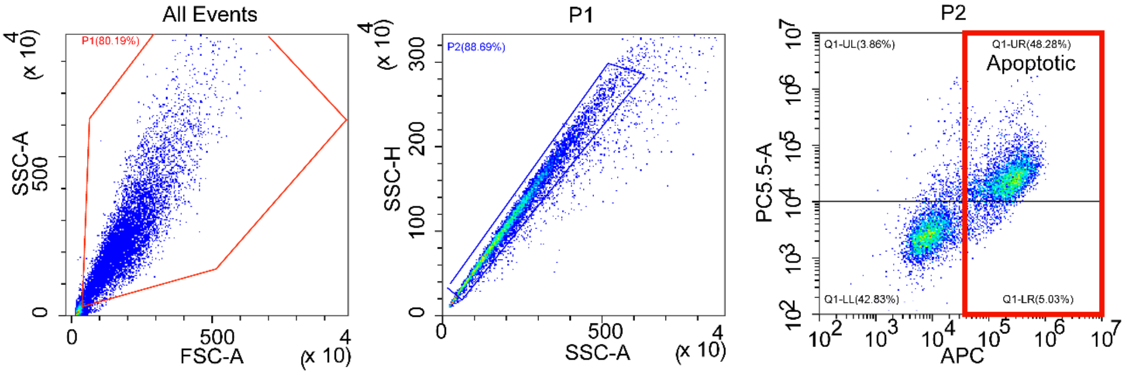

Supplementary Fig. 9 Gating strategy for all annexin V-APC/7-AAD apoptosis assay measured by flow cytometry.

Supplementary Methods

Details for Patients and Tissues

Eligible patients were 18 years or older with biopsy-confirmed hepatocellular carcinoma (not suitable for curative surgery, or local ablation). Other eligibility criteria were as follows: Child-Pugh A class liver function, an Eastern Cooperative Oncology Group performance status of 0 to 2, no previous treatment for hepatocellular carcinoma, at least 1 measurable lesion according to Response Evaluation Criteria in Solid Tumors (RECIST) version 1.1 and adequate organ function (white blood cell count ≥3.0 × 109/L, absolute neutrophil count ≥1.5 × 109/L, platelet count ≥75 × 109/L, aspartate transaminase and alanine transaminase≤5 × upper limit of the normal, creatinine clearance rate of ≤1.5 × upper limit of the normal, and left ventricular ejection ≥45%). The exclusion criteria included hepatic decompensation, including esophageal or gastric variceal bleeding or hepatic encephalopathy; central nervous system metastases; known medical history of HIV infection; pregnancy or breastfeeding; and other invasive malignant diseases. All recruited patients with hepatitis B virus–related hepatocellular carcinoma received preemptive antiviral therapy.

CRISPR-mediated PRMT3 knockout

The gRNAs for PRMT3 knockout were designed using the MIT online tool CRISPRPICK[1]. The forward and reverse primers with 20 bp target sequence and inserted into the lentiCRISPRv2GFP using BsmBI sticky ends, respectively. HEK293T cells were seeded in 10 cm plate and transfected with 10 mg lentiCRISPRv2GFP-PRMT3-KO or lentiCRISPRv2 control plasmids, 5 μg psPAX2 and 2.5 μg pVSV-G plasmids using Lipofectamine 2000 to produce lentivirus. The supernatants containing

lentivirus were harvested, filtered, and used to infect HCCs 48 to 72 hours post-transfection. After 72 h of transduction, HCC cells were subjected to 2 μg/mL puromycin (Gibco; California, USA) selection for several days.

Dot blot assay.

Firstly, mRNA (200 ng for each group) was isolated and denatured by heating at 95 °C for 3 min. Then mRNA was diluted and spotted on an Amersham Hybond-N + membrane optimized for nucleic acid transfer (GE Healthcare). The membrane was crosslinked under UV light. After the membrane was blocked with 5% nonfat milk in PBST for 1 h, it was incubated with anti-m6A antibody (Synaptic Systems, 202003, 1:2000 dilution) overnight at 4 °C. Then, the membrane was incubated with anti-rabbit IgG (Cell Signaling Technology, 7074S, 1:3000 dilution) at room temperature for 1 h. The blots were detected using the ECL detection kit (Millipore). The same mRNAs were spotted on the membrane, stained with 0.02% methylene blue in 0.3 M sodium acetate (pH 5.2) for 2 h and washed with RNase-free water for 30 mins.

Lentiviral production and infection

Lentiviral constructs expressing shPRMT3 and cDNAs for full-length PRMT3, IGF2BP1, and their mutants were transfected into HEK293T using Lipofectamine 2000 (Life Technologies) in accordance to the manufacturer's instructions. For the gene rescue experiments, shRNA targeting 30UTR of HEG1 (**Supplementary Table 3**) was used for knockdown. The supernatants containing lentivirus were collected at 48 and 72 h after transfection and used to infect target cells with polybrene (6.0 mg/ml, GeneCopoeia, Rockville, USA). After 72 h of transduction, were subjected to 2 mg/mL

puromycin (Gibco; California, USA) selection for four days.

Mass spectrometry analysis of PRMT3 interaction and IGF2BP1 arginine methylation

To identify proteins that bind to PRMT3, the immune complexes were resolved by SDS-PAGE and stained with Coomassie brilliant blue. For IGF2BP1 arginine methylation, IGF2BP1 was immunoprecipitated with anti-IGF2BP1 antibody. The immune complexes were resolved by SDS-PAGE and stained with Coomassie brilliant blue. The peptides were extracted and evaporated for liquid chromatography-mass spectrometry (LC-MS) analysis at the FITGENE company (Guangzhou, China).

IHC analysis of clinical HCC specimens

IHC analyses were performed, and the percentage of positively stained cells was quantified and statistically analyzed as previously described. Briefly, based on the IHC scores, we then dichotomized these patients as negative group, 0–25%; weak group, 25–50%; moderate group, 50–75%; strong group, 75–100%. The high-expression group is defined as patients with tumors of moderate or strong intensities, while the low-expression group is defined as patients with tumors of negative or weak intensities.

RNA-Seq

Total RNA was extracted by using Trizol and used for RNA sequencing. RNA quantification was performed with Qubit 3.0 (Thermo Fisher, MA, USA). Library preparation was performed by Epibiotek (Guangzhou, China). Briefly, total RNA was treated to remove ribosomal RNA by using GeneRead™ rRNA Depletion Kit（Qiagen, Hilden, Germany, Cat No. 180211）. Ribosome-depleted RNA was fragmented and

then used for constructing strand-specific RNA libraries by using VAHTS Stranded RNA-seq Library Prep Kit for Illumina (Vazyme, Nanjing, China, Cat. No NR602) according to manufacturer instructions. Library quality was determined on Qseq100 Bio-Fragment Analyzer（Bioptic, Taiwan, China）. The strand-specific libraries were sequenced on Illumina Novaseq 6000 system with paired-end 2×150 bp read length.

Assessment of gene editing efficiency

Gene editing efficiencies were assessed by Sanger sequencing and next-generation sequencing (NGS) using a two-step PCR-based method. Briefly, genomic DNA from cells was extracted with the DNeasy Blood and Tissue Kit (QIAGEN) and amplified for first-step PCR with Q5 High-Fidelity Polymerase (NEB). Target sequences in PRMT3 loci and primer sequences carrying 5' Illumina sequencing adaptors for PCR amplification are provided in Supplementary Table 13. PCR products were purified using QIAquick PCR Purification Kit (QIAGEN) and serve as template for second-step PCR with primer sequences carrying Illumina barcodes by Q5 High-Fidelity Polymerase (NEB). The PCR products were sequenced by ABI3730XL and Illumina Novaseq 6000 system.

Supplementary Table 1. Antibodies included in the study

Antibodies	IDENTIFIER	Catalogue No.	Lot No.	Host species	Species	Application and dilution
Anti-β-Actin	Absin	Abs830031ss	#0N18	Mouse	Hu, Mo	WB (1:1000)
Anti-GAPDH	Proteintech	60004-1-Ig	21002053	Mouse	ALl	WB (1:2000)
Anti-PRMT3	Abcam	Ab191562	1001885-4	Rabbit	Hu	WB (1:2000), IHC (1:100), IF (1:100), IP (1:50)
Anti-FLAG	Cell Signaling Technology	#14793	7	Rabbit	All	
Anti-HEG1	Bioss	bs-15449R	BJ06287323	Rabbit	All	WB (1:1000), IP (4ug)
Anti-IGF2BP1	Proteintech	22803-1-Ap	00045768	Rabbit	Hu, Mo, Rat	WB (1:750)
Anti-IGF2BP1	Santa Cruz Biotechnology	Sc-166344	K242	Mouse	Ho, Mo, Rat	WB (1:1000), IP (4ug), RIP (4ug)
Anti-ADMA	Cell Signaling Technology	13522S	4	Rabbit	All	IF (1:500)
Anti-Ki67	Abcam	Ab15580	GR3317236-1	Rabbit	Ho, Mo	WB (1:1000)
Anti-Cleaved Caspase-3	Cell Signaling Technology	#9664	45	Rabbit	Hu, Mo, Rat	IHC (1:1000)
Alexa Fluor ^{R} 488 goat anti-mouse IgG (H+L)	Thermo Fisher Scientific	# A-11001	1869589	Goat	Mo	IHC (1:2000)<br>IF (1:200)
Alexa Fluor^{TM} 594 goat anti-rabbit IgG (H+L)	Thermo Fisher Scientific	# R37117	2433881	Goat	Rat	IF (1:200)
Anti-mouse IgG	Cell Signaling Technology	7076S	36	Hr	Mo	
Anti-rabbit IgG	Cell Signaling Technology	7074S	25	Hr	Mo	WB (1:3000)
HRP Rabbit/Mouse	DAKO	K5007	41336853	NA	Rat, Mo	WB (1:3000)<br>IHC (NA)
Anti-m6A	Synaptic Systems	202003	3-123	Rabbit	Hu, Mo, Rat	Dot Blot (1:2000)
Rabbit IgG	Proteintech	B900610	20010170	Rabbit	All	IP (2ug)

Supplementary Table 2. The primers used in present study

Primer names	Forward (5'-3')	Reverse (5'-3')
PRMT3	CACTGTCTGCTGAAGCCGCATT	GTAGATGACGAGCAGGTTCTGAC
IGF2BP1	CTTTGTAGGGCGTCTCATTGGC	CCTTCACAGTGATGGTCCTCTC
HEG1	CTGCCACCTTTGCTGTTCAGA	CTGGTGTTGTCTGCGACGCATT
ACTB	CACCATTGGCAATGAGCGGTTC	AGGTCTTTGCGGATGTCCACGT

Supplementary Table 3. Sequences of RNA Oligonucleotides

Name	Sense strand/sense primer (5'-3')	Antisense strand/antisense primer (5'-3')
**siRNA**		
siPRMT3-1	GCAUGAAGAAAGCAGUUAUTT	AUAACUGCUUUCUUCAUGCTT
siPRMT3-2	GCUACCGAGAUUUCAUAUATT	UAUAUGAAAUCUCGGUAGCTT
siIGF2BP1	CCAAAGUUCGUAUGGUUAUTT	AUAACCAUACGAACUUUGGTT
siHEG1-1	CCUCUGUGCAUCUACUAAATT	UUUAGUAGAUGCACAGAGGTT
siHEG1-2	GAGUCCACCAAAGCUGUAATT	UUACAGCUUUGGUGGACUCTT
**shRNA**		
shHEG1	CCGGGATCTCAGAGGCGGATCTTTACTCGAGTAAAGATCCGCCTCTGAGATCTTTTTG	AATTCAAAAAGATCTCAGAGGCGGATCTTTACTCGAGTAAAGATCCGCCTCTGAGATC
shIGF2BP1	CCGGCCAGGAATAAAGGCTTTGTTTCTCGAGAAACAAAGCCTTTATTCCTGGTTTTTG	
**sgRNA**		
sgPRMT3-1	GGCTGATAGTGCAAATGTCA	TGACATTTGCACTATCAGCC
sgPRMT3-2	GAATTCATGTACTCAACTGT	ACAGTTGAGTACATGAATTC
sgNC	GACCGGGGCGAGGAGCTGTTCACCG	CGGTGAACAGCTCCTCGCCCCGGTC

Reference

1 Lee, H., Chang, H. Y., Cho, S. W. & Ji, H. P. CRISPRpic: fast and precise analysis for CRISPR-induced mutations via prefixed index counting. *NAR genomics and bioinformatics* **2**, lqaa012, doi:10.1093/nargab/lqaa012 (2020).
