# Supplementary material for: PRMT3-mediated arginine methylation of IGF2BP1 promotes oxaliplatin resistance in liver cancer
Source: Nat Commun. 2023 Apr 6;14:1932. doi: 10.1038/s41467-023-37542-5 (PMC10079833; doi:10.1038/s41467-023-37542-5)
Supplement: Supplementary file 13 — Description of Supplementary Files [file 41467_2023_37542_MOESM13_ESM.pdf]

Description of Additional Supplementary Files

File Name: Supplementary Data 1
Description: All differentially upregulated genes identified by CRISPR/Cas9 activation library screen.

File Name: Supplementary Data 2
Description: All differentially upregulated genes identified by transcriptome sequencing of clinical specimens responsive or non-responsive to OXA-based HAIC.

File Name: Supplementary Data 3
Description: Pathways which were activated in non-responsive tumors.

File Name: Supplementary Data 4
Description: All the read counts from the CRISPR screen data.

File Name: Supplementary Data 5
Description: Proteins detected by IP in HCC cell lysates followed mass spectrometry.

File Name: Supplementary Data 6
Description: Protein modifications detected by IP in HCC cell lysates followed mass spectrometry.

File Name: Supplementary Data 7
Description: m6A-modified transcripts identified by MeRIP (m6A)-sequencing.

File Name: Supplementary Data 8
Description: Significantly differentially expressed genes identified in PRMT3-KD cells compared to control cells.

File Name: Supplementary Data 9
Description: mRNA detected by RIP with IGF2BP1 antibody in PLC-8024 cell lysates.

File Name: Supplementary Data 10
Description: Significantly differentially expressed genes identified in IGF2BP1-KD cells compared to control cells.
