# Supplementary material for: PRMT3-mediated arginine methylation of IGF2BP1 promotes oxaliplatin resistance in liver cancer
Source: Nat Commun. 2023 Apr 6;14:1932. doi: 10.1038/s41467-023-37542-5 (PMC10079833; doi:10.1038/s41467-023-37542-5)
Supplement: Supplementary file 14 — Reporting Summary [file 41467_2023_37542_MOESM14_ESM.pdf]

nature portfolio

Corresponding author(s): Guocan Wang, Yunfei Yuan, Binkui Li

Last updated by author(s): Feb 28, 2023

Reporting Summary

Nature Portfolio wishes to improve the reproducibility of the work that we publish. This form provides structure for consistency and transparency in reporting. For further information on Nature Portfolio policies, see our Editorial Policies and the Editorial Policy Checklist.

Statistics

For all statistical analyses, confirm that the following items are present in the figure legend, table legend, main text, or Methods section.

n/a	Confirmed	
☐	☒	The exact sample size (*n*) for each experimental group/condition, given as a discrete number and unit of measurement
☐	☒	A statement on whether measurements were taken from distinct samples or whether the same sample was measured repeatedly
☐	☒	The statistical test(s) used AND whether they are one- or two-sided<br>*Only common tests should be described solely by name; describe more complex techniques in the Methods section.*
☒	☐	A description of all covariates tested
☒	☐	A description of any assumptions or corrections, such as tests of normality and adjustment for multiple comparisons
☐	☒	A full description of the statistical parameters including central tendency (e.g. means) or other basic estimates (e.g. regression coefficient) AND variation (e.g. standard deviation) or associated estimates of uncertainty (e.g. confidence intervals)
☐	☒	For null hypothesis testing, the test statistic (e.g. *F*, *t*, *r*) with confidence intervals, effect sizes, degrees of freedom and *P* value noted<br>*Give P values as exact values whenever suitable.*
☒	☐	For Bayesian analysis, information on the choice of priors and Markov chain Monte Carlo settings
☒	☐	For hierarchical and complex designs, identification of the appropriate level for tests and full reporting of outcomes
☒	☐	Estimates of effect sizes (e.g. Cohen's *d*, Pearson's *r*), indicating how they were calculated

Our web collection on statistics for biologists contains articles on many of the points above.

Software and code

Policy information about availability of computer code

Data collection	Flow cytometry data was analyzed using CytExpert (V2.4).
Data analysis	Flow cytometry<br>All flow cytometry analysis was conducted on CytoFlex (Beckman), and the data was analyzed using CytExpert (V2.4).<br>Pathway analysis<br>Pathway analysis was conducted with DAVID website<br>Statistical analysis<br>Data were analyzed using GraphPad Prism 8 software. Unpaired Student's t-test was used to analyze differences between two groups. Comparisons among multiple groups were analyzed using one-way ANOVA. The results are presented as means ± Standard Error of Mean. All boxplots indicate median (center), 25th and 75th percentiles (bounds of box), and minimum and maximum (whiskers). P < 0.05 was considered statistically significant.

For manuscripts utilizing custom algorithms or software that are central to the research but not yet described in published literature, software must be made available to editors and reviewers. We strongly encourage code deposition in a community repository (e.g. GitHub). See the Nature Portfolio guidelines for submitting code & software for further information.

Data

Policy information about availability of data

All manuscripts must include a data availability statement. This statement should provide the following information, where applicable:

- Accession codes, unique identifiers, or web links for publicly available datasets
- A description of any restrictions on data availability
- For clinical datasets or third party data, please ensure that the statement adheres to our policy

The sequence data generated in this study have been deposited in the GEO database under the accession number GSE206500 (https://www.ncbi.nlm.nih.gov/geo/query/acc.cgi?acc=GSE206500), GSE206501 (https://www.ncbi.nlm.nih.gov/geo/query/acc.cgi?acc=GSE206501), GSE206502 (https://www.ncbi.nlm.nih.gov/geo/query/acc.cgi?acc=GSE206502), GSE206503 (https://www.ncbi.nlm.nih.gov/geo/query/acc.cgi?acc=GSE206503), and GSE206504 (https://www.ncbi.nlm.nih.gov/geo/query/acc.cgi?acc=GSE206504). The remaining data are available within the Article, Supplementary Information or Source Data file. Source data are provided with this paper.

Human research participants

Policy information about studies involving human research participants and Sex and Gender in Research.

Reporting on sex and gender	Male and female
Population characteristics	Eligible patients were 18 years or older diagnosted with hepatocellular carcinoma, Child-Pugh A class liver function, an Eastern Cooperative Oncology Group performance status of 0 to 2, no previous treatment for hepatocellular carcinoma, at least 1 measurable lesion according to Response Evaluation Criteria in Solid Tumors (RECIST) version 1.1 and adequate organ function (white blood cell count ≥3.0 × 109/L, absolute neutrophil count ≥1.5 × 109/L, platelet count ≥75 × 109/L, aspartate transaminase and alanine transaminase≤5 × upper limit of the normal, creatinine clearance rate of ≤1.5 × upper limit of the normal, and left ventricular ejection ≥45%). Patients were all treated with HAIC.
Recruitment	Tissue samples for screening were prospectively obtained from HCC patients who received HAIC at the Sun Yat-sen University Cancer Center, Guangzhou, China, from 2020 to 2021. Samples were divided into Response and Non-Response groups after HAIC treatment evaluated by mRECIST criterion. Thirty-six tissue samples for efficacy prediction were retrospectively obtained from HCC patients who received HAIC followed by surgical resection at the Sun Yat-sen University Cancer Center from 2015 to 2018. From July 16 2020 until June 2 2021, we enrolled 32 patients diagnosed with advanced HCC at the Sun Yat-sen University Cancer Center for the prospective study. Tissue samples were prospectively obtained from HCC patients who received HAIC through needle biopsy. Written informed consent was obtained from each patient.
Ethics oversight	the Ethics Committee of Sun Yat-Sen University Cancer Center

Note that full information on the approval of the study protocol must also be provided in the manuscript.

Field-specific reporting

Please select the one below that is the best fit for your research. If you are not sure, read the appropriate sections before making your selection.

[x] Life sciences [ ] Behavioural & social sciences [ ] Ecological, evolutionary & environmental sciences

For a reference copy of the document with all sections, see nature.com/documents/nr-reporting-summary-flat.pdf

Life sciences study design

All studies must disclose on these points even when the disclosure is negative.

Sample size	No statistical methods were used to predetermine sample size. Sample size are based on a lot of previous publications and our previous experience, which is the most optimal to generate statistically significant results. All in vitro experiments were carried out at least three times. For in vivo experiment, cohorts of 6-7 were used (stated in the figure legend) based on previous experiments
Data exclusions	No data were excluded from the analysis.
Replication	All the experiments were replicated. Three independent experiments were carried out and each experiment was performed with at least three repeats.
Randomization	All cells and the animals were randomly allocated to experimental groups. Cells were allocated into sg-PRMT3 group and sg-NC group randomly. BALB/c nude mice were allocated into treatment group and control group randomly. Patients involved in this study were not divided randomly.
Blinding	For other experiments, the investigators were not blinded to group allocation, because the experimental design was complicated, the researchers were limited, and blinding feasibility was poor.

Reporting for specific materials, systems and methods

We require information from authors about some types of materials, experimental systems and methods used in many studies. Here, indicate whether each material, system or method listed is relevant to your study. If you are not sure if a list item applies to your research, read the appropriate section before selecting a response.

Materials & experimental systems

n/a	Involved in the study
☐	☒ Antibodies
☐	☒ Eukaryotic cell lines
☒	☐ Palaeontology and archaeology
☐	☒ Animals and other organisms
☒	☐ Clinical data
☒	☐ Dual use research of concern

Methods

n/a	Involved in the study
☒	☐ ChIP-seq
☐	☒ Flow cytometry
☒	☐ MRI-based neuroimaging

Antibodies

Antibodies used

Anti-β-Actin (WB) (Absin, Abs830031ss, #0N18); Anti-GAPDH (WB) (Proteintech, 60004-1-Ig, 21002053); Anti-PRMT3 (WB, IHC, IF, IP) (Abcam, Ab191562, 1001885-4); Anti-FLAG (WB, IP)" (Cell Signaling TECHNOLOGY, #14793, 7); Anti-HEG1(WB, IHC) (Bioss, bs-15449R, BJ06287323); Anti-IGF2BP1 (WB, IHC, IP, RIP)" (Proteintech, 22803-1-Ap, 00045768); Anti-IGF2BP1 (IF) (SANTA CRUZ BIOTECHNOLOGY, Sc-166344, K242); Anti-ADMA (WB) (Cell Signaling TECHNOLOGY ,13522S, 4); Alexa Fluor R 488 goat anti-mouse IgG (H+L) (Thermo Fisher Scientific, #A-11008); Alexa FluorTM 594 goat anti-rabbit IgG (H+L) (Thermo Fisher Scientific, #R37117); Anti-mouse IgG (WB) (Cell Signaling TECHNOLOGY, 7076S, 36); Anti-rabbit IgG (WB) (Cell Signaling TECHNOLOGY , 7074S, 25); HRP RABBIT/MOUSE (IHC) (DAKO, K5007, 41336853); Anti-N6-methyladenosine (m6A) (WB) (Synaptic Systems, #202003, K2420); Rabbit IgG (IP) (Proteintech, B900610, 20010170)

Validation

Anti-β-Actin (WB) (Absin, Abs830031ss, #0N18) is validated in the manuscript in HepG2 cells and has been cited in many manuscripts.
Anti-GAPDH (WB) (Proteintech, 60004-1-Ig, 21002053) is validated in the manuscript in PLC-8024, Huh7 and HepG2 cells and validated by the manufacturer in several cell lines.
Anti-PRMT3 (WB, IHC, IF, IP) (Abcam, Ab191562, 1001885-4) is validated in the manuscript for western blot assay, IP assay, IF assay and IHC assay, and also be validated by the manufacturer (Abcam).
Anti-FLAG (WB, IP)" (Cell Signaling TECHNOLOGY, #14793, 7) is validated in the manuscript in PLC-8024 cells and validated by the manufacturer in HEK293 cells.
Anti-HEG1(WB, IHC) (Bioss, bs-15449R, BJ06287323) is validated in the manuscript in HepG2 cells for siRNAs transfection.
Anti-IGF2BP1 (WB, IHC, IP, RIP)" (Proteintech, 22803-1-Ap, 00045768) is validated in the manuscript in PLC-8024, Huh7 and HepG2 cells for WB, IHC, IP, RIP assays and validated by the manufacturer in sh-IGF2BP1 transfected Jurkat cells. It has been cited in many manuscripts.
Anti-IGF2BP1 (IF) (SANTA CRUZ BIOTECHNOLOGY, Sc-166344, K242) is validated in the manuscript in PLC-8024 and Huh7 cells as well as HCC tissues for IF assay. It was validated by the manufacturer in Hela cells.
Anti-ADMA (WB) (Cell Signaling TECHNOLOGY ,13522S, 4) is validated in the manuscript for western blot assay, and also be validated by the manufacturer in MCF7 cells.
Anti-N6-methyladenosine (m6A) (WB) (Synaptic Systems, #202003, K2420) is validated in the manuscript for Dot blot assay in PLC-8024, Huh7 cells, and has been extensively validated by the field.
Rabbit IgG (IP) (Proteintech, B900610, 20010170) is validated in the manuscript for IP assay in PLC-8024, Huh7 and HepG2 cells, and has been extensively validated by the field.

Eukaryotic cell lines

Policy information about cell lines and Sex and Gender in Research

Cell line source(s)

Human HCC cell lines, PLC-8024 (JNO-206), Huh7 (JNO-22049) and HepG2 (JNO-10-14-3), were purchased from the Guangzhou jenniobio Biotechnology with STR (short tandem repeat) appraisal certificates.

Authentication

All the cells were authenticated using short-tandem repeat (STR) profiling.

Mycoplasma contamination

All cell lines were tested negative for mycoplasma contamination.

Commonly misidentified lines
(See ICLAC register)

No commonly misidentified cell lines were used in the study.

Animals and other research organisms

Policy information about studies involving animals; ARRIVE guidelines recommended for reporting animal research, and Sex and Gender in Research

Laboratory animals	Male, four-week-old, BALB/c nude mice were purchased from the Beijing Vital River Laboratories Animal Technology. All mice were kept under specific-pathogen free conditions in Animal Facility of Sun Yat-sen University Cancer Center. They were kept in an animal room with a 12-hour light-dark cycle at a temperature of 20-22 °C with 40-70% humidity.
Wild animals	No wild animals were used in the study.
Reporting on sex	Male
Field-collected samples	No field collected samples were used in the study.
Ethics oversight	All animal procedures were approved by Institutional Animal Care and Use Committee (IACUC) of Sun Yat-sen University Cancer Center.

Note that full information on the approval of the study protocol must also be provided in the manuscript.

Flow Cytometry

Plots

Confirm that:

- [x] The axis labels state the marker and fluorochrome used (e.g. CD4-FITC).
- [x] The axis scales are clearly visible. Include numbers along axes only for bottom left plot of group (a 'group' is an analysis of identical markers).
- [x] All plots are contour plots with outliers or pseudocolor plots.
- [x] A numerical value for number of cells or percentage (with statistics) is provided.

Methodology

Sample preparation	For the apoptosis assay, cells were treated with oxaliplatin (40 µM) for 48 hours. The cells were labeled with Annexin V/APC and 7-AAD (KeyGEN BioTECH, China) according to the manufacturer's instructions
Instrument	All flow cytometry analysis was performed on CytoFlex (Beckman).
Software	FlowJo software and CytExpert software was used to analyze the flow cytometry data.
Cell population abundance	No cell sorting was conducted in the study.
Gating strategy	The gating strategy that was used is presented in the Supplementary Figure 8

- [x] Tick this box to confirm that a figure exemplifying the gating strategy is provided in the Supplementary Information.
